# Data-Based Radiation Oncology: Design of Clinical Trials in the Toxicity Biomarkers Era

**DOI:** 10.3389/fonc.2017.00083

**Published:** 2017-04-27

**Authors:** David Azria, Ariane Lapierre, Sophie Gourgou, Dirk De Ruysscher, Jacques Colinge, Philippe Lambin, Muriel Brengues, Tim Ward, Søren M. Bentzen, Hubert Thierens, Tiziana Rancati, Christopher J. Talbot, Ana Vega, Sarah L. Kerns, Christian Nicolaj Andreassen, Jenny Chang-Claude, Catharine M. L. West, Corey M. Gill, Barry S. Rosenstein

**Affiliations:** ^1^Department of Radiation Oncology, Radiobiology Unit, Biometric and Bio-informatic Divisions, Montpellier Cancer Institute (ICM), IRCM, INSERM U1194, Montpellier, France; ^2^Department of Radiation Oncology, Maastricht University Medical Centre, MAASTRO Clinic, Maastricht, Netherlands; ^3^Radiation Oncology, KU Leuven, Leuven, Belgium; ^4^Patient Advocate, Manchester, UK; ^5^Department of Epidemiology and Public Health, University of Maryland School of Medicine, Baltimore, MD, USA; ^6^Department of Basic Medical Sciences, Ghent University, Ghent, Belgium; ^7^Prostate Cancer Program, Fondazione IRCCS Istituto Nazionale dei Tumori, Milan, Italy; ^8^Department of Genetics, University of Leicester, Leicester, UK; ^9^Fundacion Publica Galega de Medicina Xenomica-SERGAS, Grupo de Medicina Xenomica-USC, IDIS, CIBERER, Santiago de Compostela, Spain; ^10^Department of Radiation Oncology, University of Rochester Medical Center, Rochester, NY, USA; ^11^Department of Experimental Clinical Oncology, Aarhus University Hospital, Aarhus, Denmark; ^12^Division of Cancer Epidemiology, German Cancer Research Center (DKFZ), Heidelberg, Germany; ^13^University Cancer Center Hamburg (UCCH), University Medical Center Hamburg-Eppendorf, Hamburg, Germany; ^14^Division of Cancer Sciences, University of Manchester, Manchester Academic Health Science Centre, Christie Hospital NHS Trust, Manchester, UK; ^15^Department of Radiation Oncology, Icahn School of Medicine at Mount Sinai, New York, NY, USA; ^16^Department of Genetics and Genomic Sciences, Icahn School of Medicine at Mount Sinai, New York, NY, USA

**Keywords:** trial design, patient selection, biomarkers, radiotherapy, toxicity tests

## Abstract

The ability to stratify patients using a set of biomarkers, which predict that toxicity risk would allow for radiotherapy (RT) modulation and serve as a valuable tool for precision medicine and personalized RT. For patients presenting with tumors with a low risk of recurrence, modifying RT schedules to avoid toxicity would be clinically advantageous. Indeed, for the patient at low risk of developing radiation-associated toxicity, use of a hypofractionated protocol could be proposed leading to treatment time reduction and a cost–utility advantage. Conversely, for patients predicted to be at high risk for toxicity, either a more conformal form or a new technique of RT, or a multidisciplinary approach employing surgery could be included in the trial design to avoid or mitigate RT when the potential toxicity risk may be higher than the risk of disease recurrence. In addition, for patients at high risk of recurrence and low risk of toxicity, dose escalation, such as a greater boost dose, or irradiation field extensions could be considered to improve local control without severe toxicities, providing enhanced clinical benefit. In cases of high risk of toxicity, tumor control should be prioritized. In this review, toxicity biomarkers with sufficient evidence for clinical testing are presented. In addition, clinical trial designs and predictive models are described for different clinical situations.

## Introduction

Radiotherapy (RT) is one of the leading treatment modalities in oncology, and over 50% of patients diagnosed with cancer undergo RT during their course of treatment. Although RT is primarily a local treatment, patients are exposed to a risk of toxicities in the treatment field and surrounding tissues, which may develop acutely and late. Early toxicities are defined as side effects occurring during treatment or in the first 3 months after treatment completion. Late toxicities are defined as those occurring more than 3 months following RT and could increase over time for a period of many months to years. Late toxicities often persist and can have a significant negative impact on quality of life among cancer survivors. A sequential effect between early and late toxicity is often reported.

A total of 5–10% of patients will eventually develop severe side effects with a significant impact on treatment outcome or quality of life. Based on this observation and dependent upon the prognosis that reflects the type of tumor and its stage at time of treatment, dose–volume constraints to organs-at-risk are usually chosen in order to keep the risk of developing grade 3 or higher side effects below 5% (1, 2). Due to considerable progress in cancer management in recent decades, the number of cancer survivors has dramatically increased, raising new challenges in the various phases of survivorship. Thus, posttreatment morbidity and quality of life have become a critical concern in the growing patient population (3). However, there is large patient-to-patient variability for the development of adverse outcomes following RT, in terms of both prevalence and severity. While most patients will develop toxicities within the normal range, some patients demonstrate a hypersensitive phenotype and develop severe toxicities even at standard radiotherapeutic doses.

The first example of individual variation in degree of response was described by Holthusen in 1936 (4). Numerous normal tissue complication probability (NTCP) models have since been developed, and variation in normal tissue response has been shown to follow a normal distribution with 5% of patients considered as radiosensitive (5). Identification of these patients beforehand is critical to avoid morbidity because severe toxicities in a minority of patients limit the dose that can be safely delivered to the majority of patients (6). In addition, individualized risk estimation for even mild or moderate effects would provide patients with information as to their risk for complications following treatment and could be used to select patients for interventions designed to prevent or mitigate toxicities. Thus, understanding individual variation is crucial to individualization of RT treatment planning and increased therapeutic outcomes (7).

While early toxicities might compromise treatment completion, they can usually be managed with adequate care. In contrast, late toxicities can significantly affect quality of life in survivors and may require extensive treatments to alleviate symptoms. However, acute radiation reactions are not necessarily an indicator of a predisposition for late toxicity (8). Therefore, there is a need to measure individual radiosensitivity and predict the risk of toxicity before treatment. Even though many external factors such as age, concomitant medications, or recent surgery impact on the risk of toxicity, the main determinant seems to be genetic factors (i.e., intrinsic radiosensitivity). However, it is unlikely that intrinsic radiosensitivity is the product of a single genetic alteration, and as such, it should be regarded as a complex polygenic trait (7). If a link can be found between underlying genetic variation and normal tissue susceptibility to developing toxicity, then patients could benefit from genomically guided, therapeutic individualization of their treatment: early identification of patients predicted to be at high risk for radiation-induced toxicities may benefit from either RT dose reduction or hyperfractionation. Conversely, identification of patients who are at low risk of toxicity could allow for (i) hypofractionation of the treatment plan, thereby shortening treatment time or (ii) dose escalation, which could improve tumor control (9).

Several observations support the hypothesis that clinical normal tissue radiosensitivity is influenced by genomics. However, very little is known about the genetic architecture of radiosensitivity or the specific genomic variants underlying interindividual differences in normal tissue reactions to RT in unselected cancer patients. It is considered that intrinsic radiosensitivity of a patient should be regarded as a complex trait depending on the combined effect of multiple genomic alterations (9). However, factors other than intrinsic radiosensitivity (i.e., genetically determined) will influence the risk of toxicity (e.g., radiation dose, age, and comorbidities), which highlights the need to collect and include multiple variables in studies.

Several genes involved in response to radiation injury were identified because homozygous mutations resulted in unusually severe reactions to RT (e.g., ATM). Other genes studied were known to be involved in the DNA damage response to ionizing radiation or the development of fibrosis. Most studies to date investigated single nucleotide polymorphisms (SNPs) because of their high prevalence in a population. With the rise of next-generation sequencing and genome-wide assays, genomic studies have been immensely facilitated (10). SNPs associated with radiation injury have been identified using high-throughput genotyping, in genome-wide association studies (GWASs) as well as candidate gene studies (11, 12). However, SNP discovery through GWAS requires a large number of patients to reach statistical significance, and the number of patients who exhibit severe toxicity is relatively low in clinical studies (6). In addition, careful clinical consideration is required when designing radiogenomic studies. While radiation dose is the main factor influencing toxicity, additional factors, including genomic alterations (e.g., SNP) and treatment volume, may be effect modifiers of the dose–toxicity relationship. Other clinical factors such as age, smoking habits (13, 14), or preexisting conditions (autoimmune diseases such as collagen vascular diseases) (15) may influence toxicity independently of genetic background, and so it is important that risk prediction models are not restricted to only genetic or only non-genetic factors.

It is also important to consider the future development of a test for clinical application. Rigorous methodology in choice of hypothesis, methods, and result reporting is required to allow generalization of the results (16) (see also Cancer Research UK predictive biomarker roadmap: http://www.cancerresearchuk.org/prod_consump/groups/cr_common/@fre/@fun/documents/generalcontent/cr_027486.pdf). In addition, the methodology developed for reporting tumor markers could be used for evaluating the level of evidence of prognostic normal tissue radiosensitivity markers (17–20). Based on these works, our consortium developed an 18-item checklist for reporting radiogenomic studies called STROGAR (Table 1), which should stand as reference for any new predictive biomarker development (21).

**Table 1:** **STROGAR 18-item checklist for reporting radiogenomic studies from Kerns et al. (21)**.

	Item number	Recommendations
**Title and abstract**		
Title and abstract	1	Include the primary outcome(s) and type of study [whether genome-wide association studies (GWASs) or gene-specific]; provide an informative summary of the study including study design, whether discovery or validation, sample size, main end points, and major results.
**Introduction**		
Background/rationale	2	Note if the study is a GWAS or a candidate gene/SNP study and, if candidate gene study, rationale for choice of genes/SNPs; give a general description of the study setting.
Objectives	3	Define the primary/main outcome(s) of interest; describe the overall/long-term goal of the study; note if it is a discovery, validation, or multistage study. Use terminology and definitions from National Cancer Institute biomarker study guidelines (22), where applicable.
**Methods**		
Study design	4	Specify the study design (case–control, cohort); whether data were collected under a controlled trial setting; whether data were collected retrospectively or prospectively. Report power and sample size considerations.
Patient population	5	Specify the source(s) of the patients and, if multiple sources, whether they are pooled or treated as separate cohorts; define inclusion/exclusion criteria; report whether comorbidities and medications were assessed by self-report or medical records; define methods/system used for tumor staging; describe the larger patient population from which the study sample was drawn; define how major changes in treatment protocol were handled in the analysis.
Radiation exposure	6	Specify details of radiation treatment parameters including: organ(s)-at-risk, dose–time fractionation; dose rate, target volume selection (e.g., breast + boost), dose to critical substructures, dose–volume metric used, the type of treatment and treatment setting, radiation modality (e.g., external beam vs. brachytherapy), whether single or combined treatment modalities were used, whether primary treatment or salvage therapy, imaging and planning details, ICRU recommendations followed and note relaxation of criteria, note any changes in dose or treatment protocol over the time course of enrollment and whether there were any interruptions in treatment.
Phenotype(s)	7	Specify how intrapatient or pretreatment assessment was made and whether it is accounted for in defining phenotype(s); note whether patient-reported outcomes or physician-assessed outcomes are being used to define phenotype(s); note which toxicity scoring system was used (if using a common/standard system); define the grading scales used and whether the phenotype(s) is/are defined as continuous, dichotomous or categorical; describe frequency of follow-up scheduling and diagnostic intensity; define the posttreatment time frame for assessment of toxicity outcomes; describe whether outcome(s) is/are based on a single time point or the maximum/worst time point out of a series of follow-up assessments; note if/how competing risks were handled (such as non-radiation-related manifestation of the phenotype); note any medical intervention that may influence study outcome(s).
Genotyping strategy and quality control (QC)	8	Specify DNA source and isolation methods; note the methods/platform used for genotyping; specify whether genotyping was done in one stage or multiple stages; note whether genotyping was done in more than one lab or batch, and if so, how batch effects were handled; describe methods for genotype calling and cite the algorithm used; note whether genotype calling was done for the whole study sample together or in batches; describe QC methods including concordance between duplicates, control samples, and checks for cryptic relatedness; describe methods for assessing population structure; describe SNP/CNP filtering methods including filtering on per-sample call rate, per-SNP call rate, minor allele frequency, and Hardy–Weinberg equilibrium; note whether imputation was used and, if so, describe methods.
Data analysis and statistical methods	9	Define the statistical methods and models used for association testing; cite the software and settings used; describe how censoring was handled; define model selection methods used for multivariable models; describe whether all samples are analyzed together or sequentially if the study involves multiple cohorts; for multistage studies, define methods for selecting variants to follow-up in subsequent stages; describe how missing data were handled; if multiple cohorts were included, describe data harmonization methods; note whether gene–gene interaction or gene–environment interaction was investigated; describe methods used to adjust for population structure; describe methods used to correct for multiple comparisons and/or control for risk of false-positive findings.
**Results**		
Patient characteristics	10	Report number of individuals at each stage of the study (e.g., numbers examined for eligibility, numbers confirmed eligible, included in study, completed follow-up, successfully genotyped and analyzed). Give reasons for non-participation at each stage. Give description of the included patient sample regarding demographic (e.g., age at start of therapy, sex, race/ethnicity) and clinical characteristics (e.g., site and stage of primary tumor, chemotherapy, hormone therapy), details of radiation exposure, where appropriate (e.g., type, dose, boost) and potential confounders and effect modifiers (e.g., lifestyle-related factors, comorbidities, and medications), including missing data; report length of follow-up and number of events and number of patients at risk at various follow-up times, e.g., yearly. It is recommended to include a flow diagram of patients included/excluded from the study, as proposed by the CONSORT statement.
Phenotype(s)	11	Report baseline function (if relevant); report numbers of responders and non-responders for dichotomous outcomes, descriptive statistics for quantitative outcome(s), or distributions for categorical outcomes.
Genotypes	12	Report call rates; numbers of samples and numbers of SNPs excluded on the basis of QC filters; if imputation was used, note which variants are imputed and which are genotyped directly; report genetically determined racial/ethnic groups or other population clusters; report genomic inflation factor as well as corrected genomic inflation factor after controlling for population structure.
Primary associations	13	For each SNP/CNP, report: common identifier (such as dbSNP rs number), minor allele identity and frequency, phenotype by genotype category, effect size (with 95% confidence interval) and p-value; genetic inheritance model(s) used; for multivariable analyses, report unadjusted and adjusted estimate and note which covariates were included in the model(s).
Secondary analyses	14	Report subgroup analyses and/or secondary outcomes of interest.
**Discussion**		
Key results	15	Summarize key results in the context of the study objectives given in Section “Introduction.”
Limitations	16	Discuss limitations of the study in the context of bias (noting both direction and size), confounding, sample size and power, and representativeness of study population.
Interpretation	17	Provide an overall interpretation of the findings in the context of previous clinical studies, genetic association studies, and biological studies of radiation response.
Generalizability and clinical utility	18	Comment on the potential clinical utility of the findings in the context of the patient populations to which the results may apply.

This study aims to review currently available radiogenomic assays based on level of evidence and clinical relevancy and to evaluate potential ways in which these assays might be implemented in routine clinical practice.

## Available Radiogenomic Biomarkers and Their Respective Levels of Evidence

### SNP Association Studies

The initial research performed in radiogenomics involved candidate gene studies, which focused on genes encoding proteins with known associations with pathways involved in responses to radiation, such as DNA repair processes and cell cycle checkpoint control. Although a number of positive associations were reported, these studies often did not adequately correct for multiple hypothesis testing and generally were not validated in subsequent studies, with several notable exceptions described below. More recent advances in radiogenomics research have been achieved through use of SNP microarrays and the performance of GWASs in which large numbers of SNPs across the genome have been evaluated. Using both of these approaches, several large studies have been accomplished involving a rigorous analysis for association between particular SNPs and toxicity outcomes that follow the STROGAR guidelines for reporting radiogenomic studies (Table 1) (21).

The most progress has probably been made in identifying specific SNPs associated with late toxicity following RT for prostate cancer. The first radiogenomics GWASs performed aimed to identify SNPs associated with erectile dysfunction in African-American men treated with RT for prostate cancer (23). Through this study, a SNP (rs2268363) in the FSHR gene, which encodes follicle-stimulating hormone, was identified (unadjusted *p*-value = 5.46 × 10^−8^; Bonferroni *p*-value = 0.028). In another prostate cancer study, a three-stage GWAS was conducted using discovery and replication cohorts that included the use of Standardized Total Average Toxicity (STAT) score (24) as a measure of overall toxicity, combining urinary and rectal end points. A locus encompassing the TANC1 gene was associated with STAT score for overall late toxicity (25) with an odds ratio (OR) of ~6 (combined *p*-value = 4.64 × 10^−11^). More recently, a GWAS meta-analysis was performed using data from four cohorts of men treated for prostate cancer for whom toxicity was measured at 2-year post-RT (26). Two SNPs were identified in this study that met genome-wide significance. One was rs17599026, which resides on chromosome 5q31.2 and associated with urinary frequency and characterized by an OR of 3.1 (95% confidence interval 2.1–4.7, *p* = 4.2 × 10^−8^). rs7720298, which is situated on chromosome 5p15.2, was associated with decreased urine stream with an OR of 2.7 (95% CI 1.9–3.9, *p*-value = 3.2 × 10^−8^). This SNP is located in an intronic region downstream of DNAH5 exon 30. Using a candidate gene approach, a study of more than 5,000 patients who underwent RT for either prostate or breast cancer reported an association between overall toxicity and rs1801516 in the ATM gene with ORs of 1.5 for acute and 1.2 for late toxicity (27).

Several other studies have been successful in identification of SNPs associated with the development of adverse normal tissue outcomes following RT for breast cancer. For example, a study comprising four SNPs related to the TGFβ pathway reported associations with several outcomes, including breast induration, telangiectasia, and overall toxicity (28). Significant and replicated associations with adverse outcomes following breast RT were reported for the TNF SNP rs1800629 and rs2857595, which is located 25.7 kb from rs1800629 and resides in the intergenic region between NCR3 and AIF1. Another validated study of breast cancer patients identified SNP rs1139793 in TXNRD2 associated with subcutaneous fibrosis following RT (29). A separate study used a two-stage design to investigate associations between SNPs in genes whose products are involved with responses to oxidative stress with toxicities following radiation treatment of women diagnosed with breast cancer. The rs2682585 SNP in XRCC1 (30) was found to be associated with reduced risk for skin toxicities (OR 0.77, 95% CI 0.61–0.96, *p* = 0.02) and decreased STAT scores (−0.08, 95% CI −0.15 to −0.02, *p* = 0.016).

Several candidate gene SNP studies have successfully identified and validated SNPs associated with late RT toxicity in lung cancer. It was reported in studies of patients treated with RT for non-small-cell lung cancer (NSCLC) that the HSPB1 rs2868371 SNP was associated with grade 3 or greater radiation pneumonitis (31) in both the training (*p* = 0.031) and validation sets (*p* = 0.025) and that this SNP was also associated with the development of grade 3 or greater radiation-induced esophagitis (32) in both the training (*p* = 0.045) and validation cohorts (*p* = 0.031). In addition, it was reported that the TGFB1 rs1800469 SNP was associated with a higher risk of radiation esophagitis in both the training (*p* = 0.045) and validation (0.023) sets of NSCLC patients (32).

While much work remains to be done in order to identify the many radiosensitivity SNPs that likely remain undiscovered, the studies published to date represent an important step toward development of polygenic risk models. Furthermore, the GWASs have contributed to uncovering novel radiation biology genes and pathways. Functional studies of these genes will provide important information for development of pharmacological interventions to prevent or mitigate the toxic effects of radiation on normal tissues.

### Fibroblast-Based Assays

Fibroblasts have traditionally been the gold-standard considered to be the best model of normal tissue for RT studies, given the importance of fibrosis in late effects and that these cells play a large role in the supporting cellular networks that surround tumors outside of the central nervous system. The first study of this model was conducted by Burnet et al. in 1992 (33). Since then, several studies suggested that fibroblast radiosensitivity *in vitro* could predict early toxicity risk. This association was studied in the clinical setting, in breast and head and neck cancer, where fibroblast clonogenic survival after irradiation was associated with radiation-induced toxicity in patients (34). However, to date, no prospective study has been able to demonstrate a significant association between fibroblast radiosensitivity and radiation-induced toxicity in patients (35, 36).

### Radiation-Induced Lymphocyte Apoptosis (RILA) Assay

In response to the limited success of fibroblast-based tests, lymphocyte-based assays were developed in their stead. While clonogenic assays showed promise in a prospective setting and in multivariable analysis (37), the 2-week assay time was considered a barrier to clinical implementation. Therefore, Ozsahin et al. developed an assay based on CD8+ T-lymphocyte apoptosis after *in vitro* irradiation with a single 8-Gy dose (38). While no association between lymphocyte apoptosis and early toxicities was found in multivariate analyses, CD8+ T-lymphocyte apoptosis was significantly associated with late effects in various cancers in a single-center prospective trial (39) and recently confirmed in a prospective multicenter study for late breast fibrosis (40). Furthermore, this assay has been shown to be reproducible between laboratories, making it a robust test to assess individual radiosensitivity (39, 41). As such, several prospective trials are currently assessing the clinical validity of the RILA assay in different cancer settings, such as prostate or lung cancer (42).

### Other Lymphocyte-Based Assays

As lymphocytes are a convenient model for radiation response, several other lymphocyte-based assays have been used to assess individual radiosensitivity. Of these, the most common is the γ-H2AX residual foci assay. H2AX is a protein phosphorylated upon double-strand breaks formation and is one of the earliest events that can be detected after cell irradiation. The number of γ-H2AX foci after cell damage has been extensively used to evaluate response to chemotherapy and RT (43–45). However, the association between the number of residual foci and clinical response to radiation on the patient level (either measured by toxicity or tumor response) has failed to be prospectively validated.

G2 metaphase and G0 micronuclei assays were initially used to assess chromosomal radiosensitivity and predisposition to breast cancer. Along with the γ-H2AX assay, many studies have sought to find an association between G2 metaphase and G0 micronuclei assays and radiation-induced toxicity (46). However, the G2 metaphase assay has exhibited low reproducibility. As new techniques have improved this assay, its use warrants prospective validation (47, 48). Similarly, the G0 micronuclei assay has been compared to other lymphocyte-based assays, but failed to be prospectively validated for prediction of either early or late radiation-associated toxicity (49–51).

Table 2 rates these tests according to their respective level of evidence, based on the STROGAR items and adapted from the levels of evidence proposed by Simon et al. (19).

**Table 2:** **Available assays for radiosensitivity assessment with their respective level of evidence adapted from Simon et al. (19)**.

Assay	Available studies	Level of evidence
rs17599026 and rs7720298 SNPs for prostate cancer	Meta-analysis for radiation-induced toxicity (26)	I
SNPs for breast cancer	Observational studies (28, 29)	II
SNPs for lung cancer	Observational studies (31, 32)	
RILA	Prospective multicenter study for breast cancer (40)	I
Fibroblast-based assay	Retrospective studies only (34)	IV
G2 metaphase	Retrospective studies only (47, 48)	IV
G0 micronuclei	Retrospective studies only (50)	IV
Residual γ-H2AX foci	No validation studies available (45)	IV

*SNP, single nucleotide polymorphism; RILA, radiation-induced lymphocyte apoptosis*.

*Level of evidence based on REMARK guidelines (19)*.

## Clinical Implementation

For a radiosensitivity test to have utility in the clinic, a valid alternative treatment option that permits modification of the proposed treatment based on the results of a test must be available. These interventions could be dose or fractionation alterations, addition or omission of concomitant treatments (such as chemotherapy or RT mitigators), or complete exclusion of RT in hypersensitive patients if the predicted risk of toxicity exceeds the expected benefit of RT. For these individuals, treatment with either surgery and/or chemotherapy may be considered. Overall, these interventions can be divided into four situations, based on a patient’s tumor control probability (TCP) and NTCP.

### High TCP, Low NTCP

A low risk of tumor recurrence and a low risk of radiation-induced toxicity are the ideal clinical presentation. In this situation, quality of life improvement during radiation treatment should be the main goal of any intervention.

There is no need to increase total tumor dose since local control is high with standard treatment. However, alternate fractionation, such as hypofractionation, could offer a shorter treatment course with a substantial increase in quality of life. Hypofractionation has been shown to be a valid alternative for early breast cancer radiation, with schedules decreasing from 33 to 15–16 and finally 5 fractions yielding similar results in well-selected patients (52–54). In this case, hypofractionation could cut the treatment duration by half and have a significant impact on quality of life and treatment cost (55). Furthermore, a combined analysis of the START trials for breast cancer suggests that overall treatment time might be a significant determinant of local cancer control after adjuvant whole breast RT with a lower relapse rate in the accelerated arms (56).

Similarly, several hypofractionated schedules have shown promising results in prostate cancer (most recently the CHHip and HYPRO trials), with only moderate increase in rectal toxicities (57, 58). Furthermore, when analyzed from a medico-economic point of view, hypofractionated regimens could result in improved health gains at lower cost (59).

### High TCP, High NTCP

In this case, the patient would be at increased risk for developing severe toxicity following RT, but at a low risk of tumor recurrence.

This scenario is when alternate treatment plans may be most appropriate, such as a strictly surgical treatment. For example, in low-risk prostate cancer, treatment with either surgery or RT, or even active surveillance in appropriately selected patients has demonstrated similar survival outcomes (60). However, toxicity profiles differ significantly; there is a higher risk for urinary toxicity and erectile dysfunction after surgery, but a greater incidence of rectal bleeding and fecal incontinence after RT (61). Therefore, these treatment options could be offered to the patient, who could take all of these factors into careful consideration when deciding upon the type of treatment. In addition, focal therapies could be considered for appropriately selected patients.

In the case of postoperative prostate cancer, adjuvant RT has been shown to reduce the risk of biochemical failure but without overall survival improvement (62). Thus, in highly radiosensitive patients, RT could be postponed until disease recurrence or omitted altogether.

Considering early breast cancer, postoperative RT has been shown to decrease the risk of local recurrence by 15% (63). However, mastectomy with immediate reconstructive surgery could be an alternative to breast-conserving surgery plus RT for patients at high risk for development of radiation-induced toxicity (64, 65). Of course, in this case, as in any treatment change, patient’s opinion should be taken into account in the decision-making process, as a more invasive surgery might be proposed.

Alternatively, in low risk breast cancer and elderly patients, cosmetic results could be improved by reducing radiation treatment volumes with intraoperative RT or partial breast irradiation (66, 67), while maintaining excellent tumor control (68, 69).

### Low TCP, Low NTCP

Increasing total treatment dose would be the easiest intervention for a high risk of tumor recurrence in a patient with low risk of radiation-induced toxicity.

Dose escalation has been shown to improve local control in several tumor types, such as prostate or rectal cancer, where an increase in total dose could yield a higher rate of pathological complete response after surgery (70). Several dose escalation trials (in prostate, rectum, cervix or lung cancer for example) are currently recruiting, and these patients could be ideal candidates for radiogenomic trials.

Alternatively, chemotherapy or radiosensitizers could be used to increase radiation efficacy without increasing the physical dose or overall treatment time. In head and neck cancers, for example, the hypoxic modifier nimorazole could be added to the treatment regimen to overcome tumor hypoxia in patients with low risk of radiation-induced toxicity (71, 72). Gemcitabine use in locally advanced bladder cancers also has radiosensitizer effects (73).

### Low TCP, High NTCP

This presentation is the worst-case scenario with a highly radiosensitive patient and a high risk of tumor recurrence or progression.

Since the main goal of RT is to ensure tumor control, dose deescalation cannot be offered to these patients since the need for tumor control exceeds the risk of radiation-induced toxicity.

In this case, alternate fractionation could be considered, such as a hyperfractionated regimen, which may maintain the same therapeutic ratio with decreased risk of toxicity (74). When available, stereotactic body radiation therapy could also offer a decreased risk of normal tissue complications with excellent tumor control rates (75). The use of proton or carbon ion RT could also be considered if these modalities are available. Prediction models including clinical and dosimetric parameters are currently under development (76). Individual radiosensitivity measured using the aforementioned tests should be incorporated into these predictive models (77).

When alternate fractionation schedules are not applicable, radioprotectors may reduce the risk of normal tissue toxicity while maintaining comparable tumor control rates (78). Amifostine is the only Federal Drug Administration (FDA)-approved radioprotector (79). However, severe side effects (nausea, hypotension) limit its widespread clinical use. However, patients predicted to be high risk for development of adverse outcomes following RT could be good candidates for this treatment, whose pharmacologic side effects might prove more easily manageable than severe radiation-induced toxicity.

Table 3 summarizes the different clinical situations stratified by type of cancer and the suggested interventions.

**Table 3:** **Suggested treatment adaptations based on TCP and NTCP**.

Cancer type	Suggested treatment adaptations			
	High NTCP	High NTCP	Low NTCP	Low NTCP
	Low TCP	High TCP	Low TCP	High TCP
Breast	Consider the risk of recurrence first If possible, discussion of a mastectomy ± reconstructive surgery without adjuvant RT	Consider no adjuvant RT if elderly Limit large RT fields (consider partial breast RT or IORT)	Increase treatment fields (IMC, axilla) Consider hypofractionation	Consider no adjuvant RT or IORT if elderly Consider hypofractionation and accelerated RT ± partial breast RT or IORT
Prostate	Discuss possibility of surgery RT with rectal spacer RT with transponders Discuss indication of pelvic RT Discuss interest of proton therapy	Active surveillance Focal therapy Brachytherapy RT with rectal spacer RT with transponders with reduced margins	Dose escalation (boost brachytherapy if indicated) Pelvic RT if indicated	Active surveillance Hypofractionation SBRT
Lung	Surgery if possible Discuss hyperfractionation if large volumes	Surgery Very limited SBRT in case of non-operable lesions	Dose escalation Increase nodal volume (ENI) if indicated	SBRT
Rectum Esophagus	Consider the risk of recurrence first Reduce the volume of fields if possible discuss interest of proton therapy	Involved field RT Discuss the need of RT	Dose escalation if boost indicated	Involved field RT Contact therapy
Head and neck	Consider the risk of recurrence first Reduce the volume of fields if possible discuss interest of proton therapy	Involved field RT	Dose escalation Discuss the use of radiosensitizers (e.g., nimorazole)	Involved field RT Hypofractionation
Gynecological tumors	Consider the risk of recurrence first Reduce the volume of fields if possible discuss interest of proton therapy	Involved field RT No adjuvant RT in adjuvant setting	Dose escalation	Involved field RT Hypofractionation
CNS	Consider the risk of recurrence first Proton therapy is mandatory	Involved field RT No adjuvant RT	Dose escalation	Involved field RT Hypofractionation SBRT

*TCP, tumor control probability; NTCP, normal tissue complication probability; IORT, intraoperative radiotherapy; RT, radiotherapy; IMC, internal mammary chain; SBRT, stereotactic body radiation therapy; ENI, elective nodal irradiation; CNS, central nervous system*.

## Study Design and Medico-Economic Considerations

Randomized prospective clinical trials are the gold standard for interventional studies (19). There are 10 theoretical possible designs for testing clinical utility of radiogenomics models (80). However, of these, four are most applicable to randomized trials: randomize-all, interaction or risk factor-stratified design, targeted or selection design, and the individual profile design (81).

Randomize-all is the simplest design, with patients randomized for both treatments, regardless of their prognostic group and those being studied subsequently in each treatment arm. It is the most robust design to assess an intervention, regardless of patient profile. The risk factor-stratified design enables hierarchical statistical tests, by stratifying patients according to their risk level before intervention. In the targeted design, only subjects identified as high-risk patients are randomized for intervention. This model allows studies to target a specific population with a higher statistical power, even if the accuracy of the model is low. Finally, the individual profile design enables parallel therapeutic strategies to be tested in various patient profiles with patients randomized between standard treatment and a risk profile-based strategy (81).

Nevertheless, trials of radiogenomics models should carefully follow appropriate reporting guidelines, such as STROGAR, CONSORT and REMARK in order to make large-scale validation of the results easier (18, 21, 82). Development of these tests for clinical implementation should theoretically follow region-specific guidelines, such as FDA or European Medicines Agency (16). However, not all tests can comply with every item in these guidelines, such as availability of randomized interventional studies. We consider retrospective and large prospective multicenter cohorts to be a required minimum in these cases.

As normal tissue response to radiation is a polygenic trait also affected by clinical, demographic and health behavior factors, multiparametric models should be the gold standard for predictive assays. Furthermore, given that radiosensitivity assays are predictive factors, they cannot be interpreted in an independent manner (83). For example, the RILA assay has been shown to be biased by numerous factors in breast fibrosis prediction, such as smoking habits or hormone therapy (41). A nomogram has thus been developed to incorporate effect modifiers and confounding parameters when predicting risk of radiation-induced breast fibrosis. Similar considerations apply to SNP-based predictive assays. For example, the SNP tagging the TANC1 risk locus for late toxicity in prostate cancer was shown to interact with radiation dose (25). There are likely other gene-by-environment interactions that remain to be uncovered, and inclusion of interaction terms is expected to improve performance of predictive models.

From a health-economic perspective, identification of hypersensitive patients could significantly decrease the cost of radiation-associated toxicity treatments, or even the cost of treatment in low NTCP high TCP patients eligible for accelerated regimen. There are approximately 15.5 million cancer survivors in the US, and there may be substantial costs to clinically manage the toxicities that could result from treatment of their disease (84). Cardiac complications that can develop after RT to the chest area (in breast cancer or lung cancer), for example, can be substantial. Costs associated with adverse outcomes following RT are often hard to specify, because they represent a small part of a complex disease management protocol (85). However, decreasing the rate of late toxicities will undeniably lower long-term costs of cancer survivorship.

In order to clearly quantify the economic gain from radiogenomics tests, several factors need to be considered. First, the cost of the actual test needs to be taken into account. For instance, the costs of the RILA assay or targeted SNP genotyping or gene sequencing for a limited panel are generally less than 2,000€, and the price for clinical whole genome sequencing continues to drop. Treatment costs must also be considered. In this case, treatment adaptation to the NTCP of the patient could result in significant savings: total health-care expenditures for breast cancer can be decreased by 10% with hypofractionated RT (86). The cost–utility of intervention must be assessed by comparing these costs to Quality-Assessed Life Years, in all patient groups.

Once a test has shown sufficient clinical validity, it can be used to create medical companies, such as Novagray^®^ for the RILA assay, to promote and market the test.

## Conclusion and Perspectives

A large number of tests for radiosensitivity have been investigated over the last three decades, and some have proven their validity in multicenter prospective settings. Of the many tests developed over the years, only several SNP assays and the RILA assay have shown replicated performance in the development phase.

The next step that should be undertaken is the large-scale study of these models to implement clinical use and assess cost–utility. This is being carried out in Europe through the ongoing REQUITE project, using the RILA assay, as well as other validated biomarkers (42). The RILA assay incorporated in a nomogram with the other independent factors has already proven its validity in a multicenter study on breast cancer and is currently under evaluation for other cancer types (40).

Similarly, the Radiogenomics Consortium has developed the TAILORED project to validate the concept of stratification to identify cancer patients with increased individual radiosensitivity and provide cost-effective therapeutic interventions to reduce the side effects of RT for cancer. This would allow for a personalized risk-adapted approach to provide more effective treatments.

## Author Contributions

DA participated in the design of the trial, wrote the manuscript, and coordinated the corrections of all the consortium participants. AL participated in the design of the trial, wrote the manuscript, and coordinated the corrections of all the consortium participants. SG, DD, JC, PL, MB, TW, SB, HT, TR, CT, AV, SK, CA, JC-C, CW, CG, and BR participated in writing the manuscript.

## Conflict of Interest Statement

DA participated in the NovaGray start-up creation. The other authors declare that the research was conducted in the absence of any commercial or financial relationships that could be construed as a potential conflict of interest.
